# Risk and Benefit of Different Cooking Methods on Essential Elements and Arsenic in Rice

**DOI:** 10.3390/ijerph15061056

**Published:** 2018-05-23

**Authors:** Tasila Mwale, Mohammad Mahmudur Rahman, Debapriya Mondal

**Affiliations:** 1 School of Environment and Life Sciences, University of Salford, Salford M5 4WT, UK; t.mwale@edu.salford.ac.uk; 2 Global Centre for Environmental Remediation (GCER), Faculty of Science, The University of Newcastle, Newcastle, NSW 2308, Australia; mahmud.rahman@newcastle.edu.au

**Keywords:** rice, arsenic, essential elements, cooking, recommended daily intake

## Abstract

Use of excess water in cooking of rice is a well-studied short-term arsenic removal technique. However, the outcome on the nutritional content of rice is not well addressed. We determined the benefit of different cooking techniques on arsenic removal and the associated risk of losing the essential elements in rice. Overall, we found 4.5%, 30%, and 44% decrease in the arsenic content of rice when cooked with rice-to-water ratios of 1:3, 1:6 (*p* = 0.004), and 1:10 (parboiling; *p* < 0.0001), respectively. All the essential elements (except iron, selenium, and copper) incurred a significant loss when rice was cooked using the 1:6 technique: potassium (50%), nickel (44.6%), molybdenum (38.5%), magnesium (22.4%), cobalt (21.2%), manganese (16.5%), calcium (14.5%), selenium (12%), iron (8.2%), zinc (7.7%), and copper (0.2%) and further reduction was observed on parboiling, except for iron. For the same cooking method (1:6), percentage contribution to the recommended daily intake (RDI) of essential elements was highest for molybdenum (154.7%), followed by manganese (34.5%), copper (33.4%), selenium (13.1%), nickel (12.4%), zinc (10%), magnesium (8%), iron (6.3%), potassium (1.8%), and calcium (0.5%). Hence, cooked rice as a staple is a poor source for essential elements and thus micronutrients.

## 1. Introduction

The genus *Oryza* is composed of about 25 species, cultivated in tropical and sub-tropical regions of Asia, Africa, South America, and Northern Australia and distributed almost entirely across the world [1]. In Southeast Asia, both cooked rice grains and processed rice flour are an important part of the daily diet [2]; for example, rice provides around 73% of the calorific intake for the population of Bangladesh [3]. In sub-Saharan Africa, rice consumption has increased by more than 50% in the past two decades [4]. Nigeria in particular has experienced an increase in consumption of about 10% per annum since 1970, and this has been attributed to the change in consumer choice [5]. Seifarth [6] observed a rise in rice consumption by the Nigerian population due to the ease of accessibility and multiple ways in which it can be prepared. Recently, rice consumption has also increased in Northern and Southern Europe [7], and the National Diet and Nutrition Survey (NDNS) carried out between 2008 and 2012 revealed that rice was among the cereals consumed by over 70% of the UK population, thus providing important nutrients and contributing to the diet [8,9].

A variety of factors are important in rice preparation and these govern the quality of the cooked rice. For example, the rice-to-water ratio is a significant aspect and optimal use of water in cooking involves using rice-to-water ratios of between 1:1.5 and 1:2.5 [10]. The traditional method used in Southeast Asia involves a rinsing step and cooking rice in excess water (5–6 times the weight of rice), which is later discarded [11]. In the preparation of *Jollof* rice (a popular Nigerian rice dish), excess water is used to boil the rice until a rubbery texture is achieved, similar to parboiling. Thereafter, the rice is rinsed in cold water and added to tomato sauce and ground cray fish, to be cooked to an edible state [12].

Despite being widely consumed as a source of carbohydrates, certain vitamins, minerals, and other nutrients including essential elements, rice is an important route of arsenic (As) exposure [11,13,14]. Inorganic As is a class 1 carcinogen that has been linked to multiple organ cancers, skin and vascular lesions, and many more health defects [15]. According to Hojsak et al. [16], As concentration in rice is higher than in other grains like wheat and barley. The flooded conditions in which it is grown and its ability to absorb As from the soil makes rice the most contaminated cereal compared to other crops [17]. However, the concentration of As in rice depends on various factors such as origin, variety, and cooking method. For example, rice is found to be a major source of As exposure in Southeast Asia and can become the most important route in some areas where it is cooked with naturally occurring As-contaminated water [18,19]. However, simple cooking methods can remove arsenic from the grain [20] and multiple studies suggest that use of excess water for cooking plays an important role as a short-term As removal technique, and a decrease in As of between 15 and 63% has been observed in different studies when rice is cooked with As free water [11,21,22]. However, cooking in excess water also results in the loss of nutrients including essential elements [23]. A loss of 40–75% iron (Fe) depending on the type of rice and cooking technique is reported [23].

Hence, the nutritional value of rice can depend on the cooking habit adopted by different communities in different countries. This is of particular importance in developing countries where rice is the main staple and micronutrient deficiency, sometimes referred to as ‘hidden hunger’ is prevalent [24]. The present study determines the effect of three popular rice cooking methods on As and essential elements in rice collected from UK, Sri Lanka, Myanmar, and Nigeria. The contribution of rice cooked by different methods toward the recommended daily intake (RDI) of essential elements is also investigated. To the best of our knowledge, this is the first study comparing how the benefits of cooking rice to remove As can be detrimental due to the loss of essential elements, which can significantly affect the nutritional uptake in the population of developing countries subsistent on a rice-based diet.

## 2. Materials and Methods 

Rice samples (either whole grain or polished) were collected from four different countries. Among 24 rice samples tested in this study, 11 were from Sri Lanka, 3 from Myanmar, 8 from Nigeria, and 2 (of multiple origin) were purchased from a local superstore in Manchester, UK. 

### 2.1. Rice Preparation 

Ten grams of each rice sample were weighed into a 150 mL beaker and rinsed with 45 mL of deionized (DI) water until the rinse water was clear. Washed rice was subjected to three cooking methods. Rice was cooked on a hot plate with temperature set at 385 °C. The first method, known as the contemporary technique [11] involved cooking rice in 30 mL of DI water (the 1:3 ratio) until all the water was absorbed and/or evaporated. The second method, popular in Southeast Asia and referred to as the traditional method [11], required 60 mL of DI water (the 1:6 ratio), and the residual water was discarded once the rice was cooked. During the first two methods, rice was cooked for 10 min or until it was soft to touch. The third method was a type of parboiling, commonly used in Nigeria to cook the popular rice dish known as *Jollof* rice. In this method, the washed rice was cooked in 100 mL of DI water (parboiling method, 1:10) for approximately 5 min until it was slightly tender but inedible. The residual water was then discarded.

Cooked rice samples were dried in an oven at 40 °C for 24 h and thereafter in 110 °C oven until constant weight was achieved. The dried rice grains were milled to a semi-powdered form using a mortar and pestle, packaged into resealable bags and stored in a desiccator before being shipped, for analysis to the University of Newcastle, Australia. 

### 2.2. Sample Preparation for Elemental Analysis

Rice samples were digested for the analysis of total As and other elements (Fe, calcium (Ca), cobalt (Co), copper (Cu), magnesium (Mg), manganese (Mn), molybdenum (Mo), nickel (Ni), potassium (K), selenium (Se), and zinc (Zn)) based on the protocol of Rahman et al. [25]. The determination of As and other trace metals was carried out with an Agilent 7900 (Agilent Technologies, Tokyo, Japan) inductively coupled plasma mass spectrometer (ICP-MS) coupled with an autosampler (Agilent Technologies). Major elements such as Ca, Fe, K, and Mg were analyzed using the dual view (Axial and radial) inductively coupled plasma emission spectrometer (ICP-OES, PerkinElmer Avio 200). CRM, blanks, duplicates, and continuing calibration verification (CCV) were included in each batch throughout the elemental analysis. 

### 2.3. Estimated Daily Intake (EDI) of Essential Elements and Contribution to Recommended Dietary Intake 

The EDI of each essential element from consumption of rice was calculated using Equation (1)

(1) $$\mathrm{EDI}=\frac{C_{element}\times \mathrm{IR}}{1000}$$

where *C_element_* is the concentration of an essential element (mg/kg) and IR is the ingestion rate (g d^−1^) of rice, considered to be 100 g per day according to the United States Department of Agriculture (USDA) recommendations.

(2) $$\%\cdot \mathrm{contribution}\;\mathrm{to}\;\mathrm{RDI}=\frac{\mathrm{EDI}}{\mathrm{RDI}}\times 100.$$

The percentage contribution of each element to RDI was calculated (Equation (2)) using the EDI values. The RDI values were obtained from the USDA Food and Nutrition Board, Institute of Medicine, National Academies website [26]. For a particular gender, the highest possible RDI among the different age groups (RDI varies by the age) was used in this calculation. For each essential element measured in rice, we determined the percentage contribution to the RDI for each of the three different cooking methods.

### 2.4. Data Analysis

Statistical software STATA (Special edition 11.2, StataCorp LP, College Station, LP, USA) and GraphPad InStat (version 3.1, San Diego, CA, USA) were used for the data analysis. All the results were expressed as mean and standard deviation (Std. Dev). Spearman’s rank correlation (r) was used and paired non-parametric Wilcoxon test was performed to determine whether the differences observed in the concentration of As, and the essential elements in raw and cooked rice were significant.

## 3. Results

### 3.1. Quality Control Analysis

Percentage recovery of As and other elements in the rice flour certified reference material NIST 1568b (*n* = 6) were as follows: As 110%, calcium (Ca) 107%, cobalt (Co) 101%, copper (Cu) 132%, iron (Fe) 89%, potassium (K) 88%, magnesium (Mg) 80%, manganese (Mn) 97%, molybdenum (Mo) 92%, selenium (Se) 120%, and zinc (Zn) 86%. The limit of detection (LOD) and limit of quantification (LOQ) of each element in the solution matrix are presented in Table 1 below.

### 3.2. Raw Rice

Arsenic and other essential elements in raw rice are shown in Table 2. Overall, As concentration in raw rice (*n* = 24) was found to be 0.132 ± 0.10 mg/kg, with an average concentration higher in UK rice samples (0.25 ± 0.02 mg/kg) and lowest in Nigerian rice (0.1 ± 0.097 mg/kg). Furthermore, the relationship between As and essential elements was investigated. The results revealed a significant (*p* < 0.05) positive correlation between As and Mo (r = 0.46), Mg (r = 0.49), K (r = 0.62), and Fe (r = 0.50). There was also a positive correlation between As and Ca (r = 0.38, *p* < 0.1). 

### 3.3. Effect of Cooking on As in Rice

**Figure 1:**
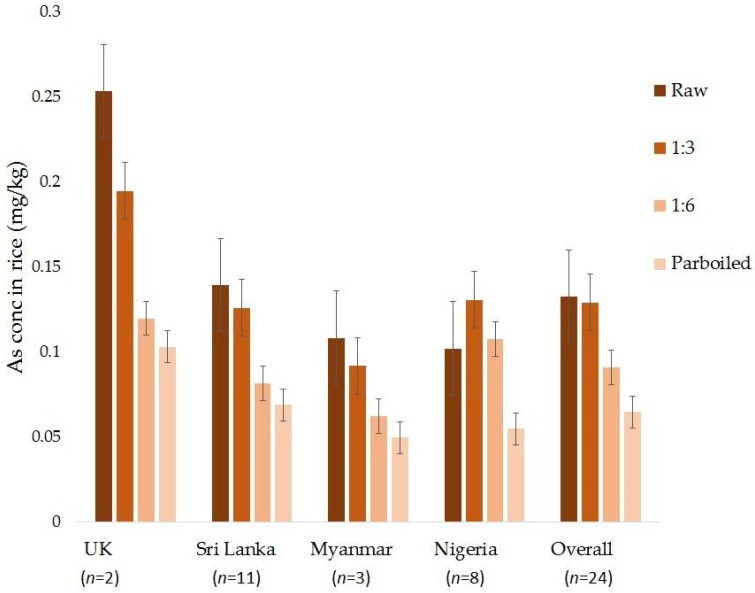
Effect of cooking technique on arsenic concentrations in rice samples collected from different countries.

Overall a 4.5%, 30%, and 44% reduction in total As was observed upon cooking rice using the three methods; 1:3, 1:6, and parboiling, respectively (Figure 1). Decrease in As was significant for 1:6 (*p* = 0.004) and parboiling (*p* < 0.0001) techniques. We found the highest reduction in arsenic content in UK rice (52%) followed by rice from Myanmar (42%), Sri Lanka (34%), and Nigeria (9%) when cooked with excess water (the 1:6 rice-to-water ratio). Nigerian raw rice samples had a wide variation in the arsenic content (min 0.01 to max 0.31 mg/kg) and the effect of cooking was not easily detected as most of the samples had very low arsenic concentrations. On parboiling (1:10, rice-to-water ratio), the maximum decrease in arsenic content occurred in UK rice (59%) followed by rice from Myanmar (52%), Sri Lanka (46%), and lastly Nigeria (33%). The differences in the loss rates of As from rice after cooking could be attributed to the different rice varieties (genotypes) apart from the variation due to different sample sizes.

### 3.4. Effect of Cooking on Essential Elements in Rice and Resultant Contribution to RDI

**Figure 2:**
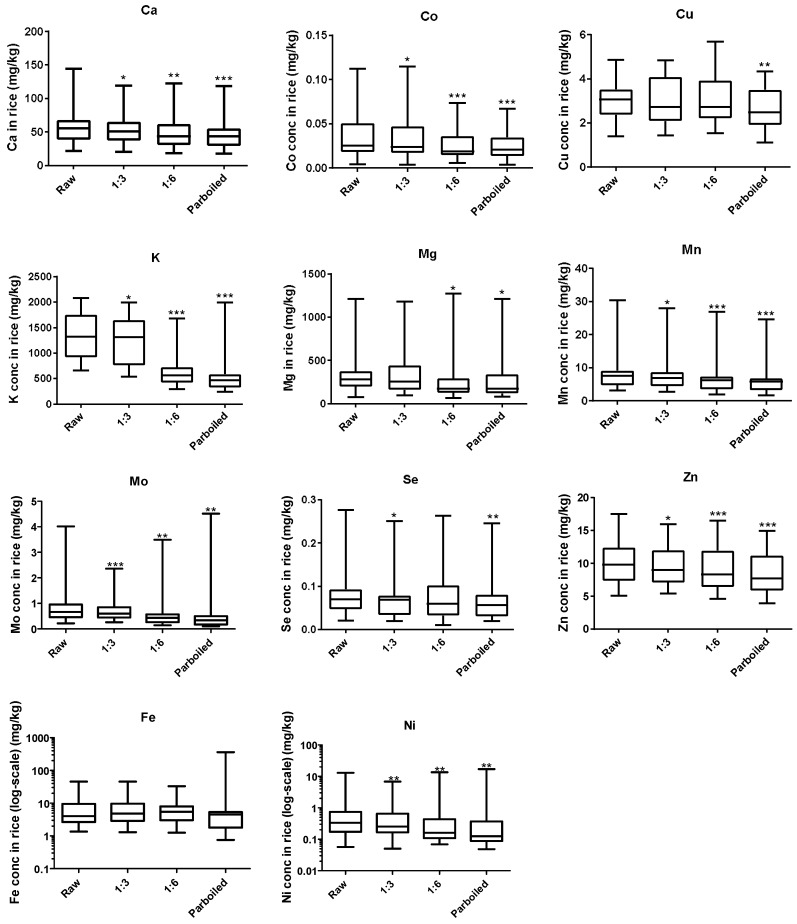
Effect of cooking technique on elemental concentrations in rice. *** *p* < 0.001, ** *p* < 0.01, * *p* < 0.05. Paired non-parametric Wilcoxon test was performed to determine the significance in raw and cooked rice. Each box represents the interquartile range (25th and 75th percentile); the band near the middle of the box is the 50th percentile (the median), the whisker represents the 5th and 95th percentile.

We found a negative correlation between the volume of cooking water and most of the essential elements in the rice samples (Figure 2). A significant reduction was observed for all the elements except Cu, Fe, and Se when rice was cooked using the 1:6 ratio and the following trend in percentage reductions was observed: K (50%) > Ni (44.6%) > Mo (38.5%) > Mg (22.4%) > Co (21.2%) > Mn (16.5%) > Ca (14.5%) > Se (12%) > Fe (8.2%) > Zn (7.7%) > Cu (0.2%). Moreover, the method used in the preparation of *Jollof* rice (parboiling) resulted in the further loss of essential elements and the percentage loss to raw rice had the following trend: K (58.9%) > Ni (52.9%) > Mo (52%) > Fe (24.4%) > Mg (23.8%) > Mn (20.8%) > Co (20.4%) > Se (19.3%) > Ca (18.9%) > Zn (14.2%) > Cu (12.5%), with significant decrease for all except Fe. Contemporary cooking (the 1:3 ratio) also resulted in the loss of essential elements but to a much lesser extent compared to 1:6 and parboiling methods. 

The contribution to RDI (Table 3) was highest for rice cooked using the 1:3 ratio followed by 1:6 and parboiling (except for Fe) and the trend for the different essential elements was Mo (154.7%) > Mn (34.5%) > Cu (33.4%) > Se (13.1%) > Ni (12.4%) > Zn (10%) > Mg (8%) > Fe (6.3%) > K (1.8%) > Ca (0.5%) for the 1:6 ratio. This trend was similar for both 1:3 and parboiling methods. 

## 4. Discussion

The Joint FAO-WHO Codex Alimentarius Commission in July 2014 established a maximum level of 0.2 mg/kg for inorganic As in polished rice [27] but in a previous study Banerjee et al. [28] reported elevated genotoxic effects in a population from West Bengal, India, consuming cooked rice with total As greater than 0.2 mg/kg. In this study, six out of 24 raw rice samples had total As greater than 0.2 mg/kg. Considering 10–90% of these could be inorganic arsenic [29], most of the rice samples had inorganic arsenic below the FAO guideline. When cooked using a rice-to-water ratio of 1:3, the most common method used in Western countries [11], though we observed an overall decrease of 4.5%, one of the Nigerian rice samples (0.27 mg/kg), three from Sri Lanka (0.24, 0.25, and 0.31 mg/kg), and one (0.22 mg/kg) out of the two UK samples had total As concentration greater than 0.2 mg/kg, the threshold observed in the Banerjee et al. study. Moreover, the Nigerian sample, which had 0.31 mg/kg of As in raw rice, when cooked using the 1:3 ratio, had 0.27 mg/kg , and had 0.23 mg/kg when cooked using the 1:6 ratio, hence not just the raw sample but the cooked rice had As concentration greater than 0.2 mg/kg. The rest of the rice samples had an As concentration of less than 0.2 mg/kg when cooked using the 1:6 ratio, with an overall decrease of 30%. Cooking rice in excess water (1:6) is known to reduce Asc content by 35% [22], 57% [11], between 15 and 50% [23], and up to 63% [21]. This traditional method is still used by more than 90% of villagers in Southeast Asian regions such as Bangladesh and the Bengal delta of India [11], one of the worst arsenic-affected areas in the world. In a recent study [23], cooking with even higher volume of water (a 1:10 rice-to-water ratio) was found to reduce total As content by about 30% for polished long and medium rice grain, 65% for parboiled and 45% for brown rice. Normally, parboiling is a treatment practiced in many Asian and African countries to gelatinize the starch of rice and can be done by different methods [30]. However, the method used in this study is usually practiced in West Africa, as mentioned earlier. While previous studies have largely reported the effect of different cooking methods on parboiled rice samples [23,31], to the best of our knowledge, this is the first study looking at the effect of parboiling to prepare *Jollof* rice on the As content of rice. Though we observed an overall 44% reduction, the lowest decrease was for Nigerian rice (33%) where this preparation is common.

Amongst all the essential elements that were analyzed in the current study, we observed a positive correlation between As and Mo, Mg, K, Fe, and Ca in raw rice. Previous studies reported similar correlations between As and K, Mg, Mn, and Fe (estimated using Table 2 and Table 3 in Pinto et al. [13]) and between As and Ni, Se, and Zn (estimated using Table 2 in Somella et al. [32]). A significant loss of elements was noted when rice was cooked using the three different methods, the concentrations essentially decreasing as the volume of cooking water increased. According to Mihucz et al. [33], the loss of essential elements was enhanced by their location on the surface of the rice grain, which makes them susceptible to easy removal through washing and cooking. Among all essential elements, the maximum loss was observed for K due to cooking. The concentration of K ranged from 661 to 2084 mg/kg in raw rice, with the highest concentration found in UK rice (1842 mg/kg, Table 2) followed by the Nigerian rice (1438 mg/kg). However, Nigerian rice samples suffered the maximum loss both after cooking with excess water (1:6; 58.3%) and parboiling (used for *Jollof* rice; 67.8%). In a recent study on mineral composition of commonly consumed local foods in Nigeria, authors reported a low K in *Jollof* rice and mentioned that K was below the recommended levels in the analyzed food samples [34]. The essential element that was least affected by cooking was Cu. The concentration of Cu in raw rice ranged from 1.39 to 4.86 mg/kg, with the highest concentration in UK rice (3.96 mg/kg, Table 2) and the lowest (2.62 mg/kg) in Sri Lankan rice.

A decrease in the contribution of essential elements to the RDI was observed with an increase in rice cooking water, except for Fe (Table 3). Overall, results revealed that Mo contributed the most and in fact more than the required amount to the RDI, 156.8%, 154.7%, and 147.9% when rice was cooked using the 1:3 1:6 ratios and the parboiling method, in spite of the fact that there was substantial decrease in concentration (9.4% for the 1:3 ratio, 38.5% for the 1:6 ratio, and 52% for the parboiling method) due to cooking. UK rice had the highest concentration (2.48 ± 2.17 mg/kg) of Mo in raw rice, whilst Myanmar rice had the lowest (0.45 ± 0.36 mg/kg). A study carried out by Lv et al. [35] on the effect of the environment (air quality, water, and rice) on a population in Zhongxiang, China, revealed that Mo in rice was one of the elements responsible for increasing human health and longevity in the surveyed population. Similarly, Ca and Se in rice were also positively correlated with longevity [35]. However, based on this study, Ca, which contributed the least to the RDI (0.55%, 0.49%, and 0.48% for males and 0.46%, 0.41%, and 0.40% for females for rice cooked with 1:3, 1:6, and parboiling methods, respectively) experienced 8.3% (the 1:3 ratio), 14.5% (the 1:6 ratio), and 19% (parboiling) reductions due to cooking, whilst Se, which was also reduced to a similar extent due to cooking (13.7%, 12%, and 19% via 1:3, 1:6, and parboiling methods, respectively) contributed around 12.4% (the 1:3 ratio), 13% (the 1:6 ratio), and 12% (parboiling) to RDI.

Micronutrients are important for the correct functioning of the body, and a lack of or any imbalance of micronutrients are associated with disease aetiology [36]. In addition, insufficient mineral intake can have an effect on our everyday activities, our behaviour, and our physical, intellectual, and emotional states [37]. Severe cases of Se and Fe deficiency are common all over the world, and low dietary intakes of Mg, Ca, and Zn exist amongst populations in multiple countries [13]. Iron deficiency is more prevalent in Southeast Asia and Africa, affecting pregnant women, children, and adolescents. Moreover, conditions occurring from micronutrient deficiencies affect over 2 billion people worldwide [38]. Based on our study, it is clear that cooked rice is a poor source of essential elements and thus micronutrients; however, consumed globally, it is the staple for more than half of the world’s population [39] and is hence a significant source of minerals, especially in certain countries such as rural India and Bangladesh, which are dependent on a rice-based diet [7,13]. According to Maclean et al. [7], micronutrient deficiencies are more severe in areas where rice is a major staple. In poor Asian communities, vegetables are the most popular accompaniments to rice because the population cannot afford, or do not have access to, other types of food, such as meat and fish, from which they obtain additional nutrients [40]. Considering a rice consumption rate of 500 g/day, [18] we found that rice cooked using the 1:6 ratio, which is the traditional method used in Southeast Asia, contributed to 2.5% of Ca, 19% and 9% of Fe for males and females, respectively, 71% and 105% of Zn for males and females, respectively, and more than 100% of required Se based on the RDI of essential elements for Southeast Asia [41]. 

The mineral content of rice (depending on the rice variety) is known to be highly influenced by the degree of rice processing such as polishing, milling [42], and parboiling [13,30], but the effect of cooking is less explored. Choice of cooked rice texture differs from one region to another. For example, Das et al. [43] highlighted the different preferences in some parts of the world, stating that countries in the west enjoy long-grain, light, fluffy or slightly dry single rice grains with flavour and no hard core, while Japanese consumers prefer short-grain sticky rice and Indians like medium-grain, light, fluffy individual grains with flavour and a soft core. Hence, methods of rice preparation differ widely. 

## 5. Conclusions

Our results show that cooking rice in excess water (1:6 and parboiling) reduces the risk of As exposure but results in a reduction of essential elements, thus increasing the risk of micronutrient deficiency, which has severe ramifications especially in children, pregnant women, and the elderly in developing countries dependent on a rice-based diet. We also found that arsenic removal and loss of essential elements due to cooking vary widely depending on the type of rice and its origin.

## Figures and Tables

**Table 1:** Limit of detection (LOD) and limit of quantification (LOQ) values for As and essential elements.

	As	Ca	Co	Cu	Fe	K	Mg	Mn	Mo	Ni	Se	Zn
	µg/L	mg/L	µg/L	µg/L	mg/L	mg/L	mg/L	µg/L	µg/L	µg/L	µg/L	µg/L
LOD	0.01	0.05	0.05	0.02	0.01	0.1	0.05	0.01	0.05	0.1	0.2	0.01
LOQ	0.03	0.17	0.17	0.07	0.03	0.3	0.17	0.03	0.17	0.33	0.67	0.03

**Table 2:** Total arsenic and concentrations of essential elements (mg/kg) in raw rice.

Location	As	Ca	Co	Cu	Fe	K	Mg	Mn	Mo	Ni	Se	Zn
United Kingdom (*n* = 2)	0.25 ± 0.02	80.64 ± 62.83	0.02 ± 0.01	3.96 ± 0.96	31.00 ± 20.49	1842 ± 342	736 ± 671	19.01 ± 16.05	2.48 ± 2.17	7.23 ± 8.10	0.07 ± 0.03	13.77 ± 5.32
Sri Lanka (*n* = 11)	0.14 ± 0.12	62.13 ± 32.41	0.04 ± 0.02	2.62 ± 0.93	4.67 ± 2.96	1285 ± 439	376 ± 271	8.24 ± 5.27	0.67 ± 0.39	0.22 ± 0.12	0.09 ± 0.04	10.00 ± 3.03
Myanmar (*n* = 3)	0.11 ± 0.03	66.35 ± 9.49	0.02 ± 0.01	3.04 ± 0.66	3.04 ± 0.62	845 ± 124	305 ± 37	7.48 ± 2.00	0.45 ± 0.36	0.20 ± 0.14	0.12 ± 0.14	12.60 ± 0.59
Nigeria (*n* = 8)	0.10 ± 0.10	45.63 ± 10.22	0.04 ± 0.04	3.51 ± 0.63	10.69 ± 12.64	1438 ± 408	247 ± 87	6.03 ± 2.34	0.81 ± 0.22	0.80 ± 0.43	0.06 ± 0.02	8.21 ± 2.71
Overall (*n* = 24)	0.13 ± 0.10	58.70 ± 27.97	0.04 ± 0.02	3.08 ± 0.90	8.67 ± 11.28	1327 ± 447	354 ± 267	8.31 ± 6.10	0.84 ± 0.75	1.00 ± 2.58	0.08 ± 0.06	10.04 ± 3.27
Range	0.01	22.11	0.004	1.39	1.36	661	77	3.08	0.22	0.06	0.02	5.07
	0.40	144.36	0.11	4.86	45.49	2084	1211	30.36	4.01	12.96	0.28	17.53

Concentrations are presented as mean ± standard deviation. Sample size is represented by ‘*n*’.

**Table 3:** Percentage contribution of cooked rice to the recommended daily intake (RDI) of essential elements when cooked using the three different methods.

			Cooking Technique		
Essential Element	Gender	RDI (mg/day)	1:3 (%)	1:6 (%)	Parboiled (%)
Ca	M	1000	0.55	0.49	0.48
	F	1200	0.46	0.41	0.4
Cu	M & F	0.9	33.8	33.4	29.5
Fe	M	8	10.9	8.8	23.8
	F	18	4.8	3.9	10.6
K	M & F	3510	3.6	1.8	1.6
Mg	M	420	8.4	7.0	6.9
	F	320	11.1	9.1	9.1
Mn	M	2.3	33.8	30.3	28.8
	F	1.8	43.2	38.7	36.9
Mo	M & F	0.045	156.8	154.7	147.9
Ni	M & F	1	6.7	12.4	14.2
Se	M & F	0.055	12.4	13.1	11.9
Zn	M	11	8.6	8.4	7.9
	F	8	11.8	11.6	10.8

Co is not included in the RDI calculation because it is not amongst the list of essential elements recommended by the USDA; M: Male; F: Female; mg/day: milligram per day.
